# An Unstructured Supplementary Service Data System to Verify HIV Self-Testing Among Nigerian Youths: Mixed Methods Analysis of Usability and Feasibility

**DOI:** 10.2196/44402

**Published:** 2023-09-25

**Authors:** David Ayoola Oladele, Juliet Iwelunmor, Titilola Gbajabiamila, Chisom Obiezu-Umeh, Jane Ogoamaka Okwuzu, Ucheoma Nwaozuru, Adesola Zaidat Musa, Kadija Tahlil, Ifeoma Idigbe, Jason Ong, Weiming Tang, Joseph Tucker, Oliver Ezechi

**Affiliations:** 1 Department of Behavioral Science and Health Education Saint Louis University Saint Louis, MO United States; 2 Clinical Sciences Department Nigerian Institute of Medical Research Lagos Nigeria; 3 Wake Forest University School of Medicine Winston-Salem, NC United States; 4 Institute of Global Health and Infectious Diseases University of North Carolina at Chapel Hill Chapel Hill, NC United States; 5 Melbourne Sexual Health Centre (MSHC) University of Melbourne Melbourne Australia; 6 Division of Infectious Diseases, Department of Medicine University of North Carolina at Chapel Hill Chapel Hill, NC United States; 7 Faculty of Infectious and Tropical Diseases London School of Hygiene and Tropical Medicine London United Kingdom

**Keywords:** adolescent, adolescents and young adults, Africa, AYA, development, feasibility, HIV self-testing, HIV, HIVST, information system, Nigeria, platform, testing, think aloud, unstructured supplementary service data, usability, user-centered, USSD, young adult, youth

## Abstract

**Background:**

Mobile health (mHealth) interventions among adolescents and young adults (AYAs) are increasingly available in African low- and middle-income countries (LMICs). For example, the unstructured supplementary service data (USSD) could be used to verify HIV self-testing (HIVST) among AYAs with poor bandwidth.

**Objective:**

The aim of this study is to describe the creation of an USSD platform and determine its feasibility and usability to promote the verification of HIVST results among AYAs in Nigeria.

**Methods:**

We developed and evaluated a USSD platform to verify HIVST results using a user-centered approach. The USSD platform guided AYAs in performing HIVST, interpreting the result, and providing linkage to care after the test. Following the usability assessment, the USSD platform was piloted. We used a mixed methods study to assess the platform’s usability through a process of quantitative heuristic assessment, a qualitative think-aloud method, and an exit interview. Descriptive statistics of quantitative data and inductive thematic analysis of qualitative variables were organized.

**Results:**

A total of 19 AYAs participated in the usability test, with a median age of 19 (IQR 16-23) years. There were 11 females, 8 males, and 0 nonbinary individuals. All individuals were out-of-school AYAs. Seven of the 10 Nielsen usability heuristics assessed yielded positive results. The participants found the USSD platform easy to use, preferred the simplicity of the system, felt no need for a major improvement in the design of the platform, and were happy the system provided linkage to care following the interpretation of the HIVST results. The pilot field test of the platform enrolled 164 out-of-school AYAs, mostly young girls and women (101, 61.6%). The mean age was 17.5 (SD 3.18) years, and 92.1% (151/164) of the participants reported that they were heterosexual, while 7.9% (13/164) reported that they were gay. All the participants in the pilot study were able to conduct HIVST, interpret their results, and use the linkage to care feature of the USSD platform without any challenge. A total of 7.9% (13/164) of the AYAs had positive HIV results (reactive to the OraQuick kit).

**Conclusions:**

This study demonstrated the usability and feasibility of using a USSD system as an alternative to mobile phone apps to verify HIVST results among Nigerian youth without smartphone access. Therefore, the use of a USSD platform has implications for the verification of HIVST in areas with low internet bandwidth. Further pragmatic trials are needed to scale up this approach.

## Introduction

Mobile health (mHealth) interventions are increasingly available in African low- and middle-income countries (LMICs) [1]. Some of its uses were documented in the promotion of maternal health, antenatal clinic attendance, HIV testing [2,3], and sexual and reproductive health (SRH) among adolescents and young adults (AYAs) in Africa [2]. The increase in its use is partly because AYAs may have more education, better bandwidth, and better access to cell phones compared to 10 years ago [4]. The use of mHealth in this population has increased the access to SRH services for AYAs, reduced the incidence of unsafe abortions, promoted contraception, and enhanced HIV self-testing (HIVST) [5-7]. And it is increasingly becoming the medium for addressing the SRH challenge among AYAs, some of which include the high prevalence of sexually transmitted infections (STIs) and HIV [8-11]. mHealth intervention has become common in Nigeria because the high HIV burden among AYAs requires innovative youth-centered implementation strategies. For example, implementation strategies to promote HIV self-testing have significantly improved access to HIVST services in Nigeria [12-14].

A major challenge with the scale-up of the HIVST services among AYAs in African LMICs is how to verify the results for individuals who self-test [15]. A recent systematic review documented various means of verifying HIVST results, including provision of supervision by health care workers, return of used test kits, and electronic transmission of photographs [15]. It then follows that the use of mobile phones for the verification of HIVST results, a form of electronic transmission, remains a feasible and acceptable intervention in this population.

However, a significant proportion of Nigerian AYAs have analog phones and low bandwidth [14]. Therefore, mHealth strategies are needed to promote HIVST among AYAs without smartphone access. The unstructured supplementary service data (USSD) platform represents a viable alternative to verify HIVST uptake in this population. The USSD is a protocol used to send text messages, similar to SMS. USSD uses character codes available on the mobile phone and creates a real time communication session between the phone and another device, usually a network or server. With USSD, users can interact directly from their mobile phone by selecting from various menus [16]. Unlike an SMS, during a USSD session, a USSD message establishes a connection in real time. This means that USSD allows 2-way information communication as long as the communication line remains open. It could provide step-by-step instructions to execute a task and collect some basic information stored on the backend [17].

The existing mHealth literature focuses on high-income countries and centers on the perspective of people with higher electronic device bandwidth [14]. Additionally, the USSD platform has been used in Africa to address some SRH issues, but to the best of our knowledge, no studies have used USSD to verify HIVST results, thereby promoting its uptake. There is a need to deliver USSD intervention to verify HIVST results among AYAs who use analog phones with limited bandwidth.

This study, therefore, described the development of a USSD platform and determined the feasibility and usability of this platform to verify HIVST results among AYAs in Nigeria.

## Methods

We designed this study as a parallel mixed methods investigation.

### Development of the Unstructured Supplementary Service Data Platform

The innovative tools to expand HIVST (I-TEST) project is a National Institutes of Health (NIH)–sponsored research collaboration between the Nigerian Institute of Medical Research (NIMR), Saint Louis University, New York University, and the University of North Carolina at Chapel Hill [18]. To meet the challenge of using mHealth to promote HIVST among young people in Nigeria who do not have access to smart mobile phones, we engaged a team of developers to develop a USSD platform with the aim of verifying HIVST uptake by guiding users (youths) through the proper use of the HIVST kit and linking them to care and anonymous counseling.

**Figure 1:**
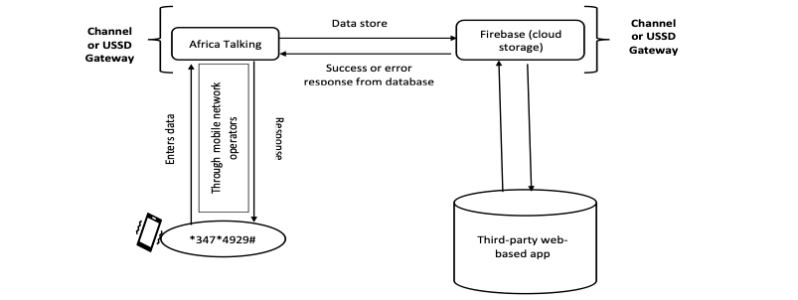
General schematic describing the unstructured supplementary service data (USSD) system.

First, the participant enters a numeric code (eg, *347*4929#) on their mobile phone. The network provider then validates the code, obtained from Africa’s Talking Ltd, a Kenya-based communication company that organizes USSD code service for a fee. After validation of the code, the process is repeated in reverse after contact with the third-party web-based app, with the response going back to the USSD gateway, which displays the response’s content on the screen of the user’s mobile phone (Figure 1).

The USSD code requires the user to enter key information required for the research, like a unique ID, the current state of residence, the current local government area of residence, age, sexual orientation, gender, and finally, the number of lines that appear on the test kit after the HIVST process has been completed. The USSD platform also provided an opportunity for posttest counseling by collecting information on participants’ mobile numbers (Multimedia Appendix 1).

On completing the test, the participant is sent an SMS notifying him or her of the test result. The information collected from the participant is saved into a database. Also, an administrative portal at the backend helps manage all records of participants involved in the research (Multimedia Appendix 2). The records on this admin portal can be exported.

### Study Setting

This study took place in Lagos State, Nigeria, which is a state located in the South-West geopolitical zone of Nigeria with an estimated 24.6 million inhabitants as of 2015. Lagos State is a major financial center in Nigeria. It has 20 local government areas and 37 local council development areas.

### Study Design

#### Usability Study

A mixed methods approach was used for the usability study to provide contextualized insights into the qualitative data and a quantitative heuristic evaluation of the user’s experience to provide a concurrent assessment of the usability of the USSD platform to verify HIVST results.

#### A Think-Aloud and an Exit Interview

During the user testing phase, a qualitative evaluation of user experience was done using the think-aloud method, and an exit interview was done for participants to document further their experience of the USSD platform for the HIVST.

Through the think-aloud method, participants performed HIVST, verbalized their experience (thinking aloud), and said whatever came into their minds when completing the task [19,20]. After completing the task, they were invited to participate in an exit interview with the research assistants for the study to document their experience further.

Study population and participants’ recruitment: the user testing was conducted among out-of-school adolescents and AYAs in Lagos State, Nigeria. The I-TEST research team from NIMR worked with adolescent peer educators working with an indigenous nongovernmental organization in Lagos to recruit the study participants. A total of 19 AYAs took part in the user testing phase. Eligibility and inclusion criteria included being aged 14-24 years old, owning a mobile phone, and being able to read. AYAs who do not have mobile phones, are currently enrolled in an educational institution, and cannot read and write were excluded from this study. AYAs were provided with oral HIV self-test kits (Orasure) and performed HIVST using USSD guidance. Oral HIVST has been shown to be effective in earlier studies [14,18].

#### Usability Heuristic Evaluation

Heuristic evaluation is the process of evaluating a mobile technology, app, or system to determine issues with its use and discover flaws. It is traditionally carried out by experts who can quickly and efficiently evaluate systems. A major setback with the assessment method is that usability experts are usually not the system’s end users. Hence, the heuristic evaluation mostly ignores the end users’ point of view on the system or app. Previous research has underlined the importance of obtaining end user feedback in the process [21,22]. In this study, we adapted Nelson’s usability heuristics to understand the usability of the USSD platform. This included how efficiently users can navigate the USSD system, perform an HIVST kit, interpret their results, and access posttest referral services. The advantage of the heuristic evaluation of the user’s experience lies in its ability to efficiently identify usability issues in an inexpensive way as multiple evaluations are done independently at the same time [19]. Specifically, users answered 21 questions through a simplified dichotomous scale that addresses various aspects of Nelson’s 10-item heuristics.

#### Data Collection for Usability Testing

Participants for the usability test were invited to an easily accessible place in the community where informed consent was obtained before the testing. The think-aloud approach [23] was used for the usability testing, which required users to continuously talk about their thoughts as they interact with the USSD platform by freely expressing what they were doing and why and stating when they encountered any problems in the process. This process was followed by the administration of the heuristic questionnaires, which the interviewer administered through an exit interview. An audio recording of the exit interview was done for further analysis.

#### Pilot Testing

The pilot study of the USSD platform in verifying HIVST was done after the usability assessment to generate preliminary real-life effectiveness data on using the platform before deployment for large-scale use. During this process, we evaluated data collected from participants who used the USSD platform in an informal settlement in Lagos, Nigeria.

#### Study Population and Participants Recruitment

The pilot testing was conducted among out-of-school and in-school AYAs in Lagos State, Nigeria.

The I-TEST research team from NIMR worked with an indigenous nongovernmental organization in Lagos, with existing collaboration with community mobilizers with previous experience in SRH research among adolescents in the community. A total of 164 AYAs were recruited during the pilot phase from the urban inner-city of Yaba in Lagos State. Inclusion criteria were being aged between 14 and 24 years, owning a mobile phone, and having the ability to read. Typically, AYAs in the community were invited to the designated areas in the community where comprehensive SRH services were provided, including counseling. They were then invited to perform HIVST using the USSD platform.

#### Data Collection for Pilot Testing

Before the pilot testing exercise, a community mobilization exercise was carried out. This involved proper community entry with the community development association (CDA) chairman and other community stakeholders who helped secure a space in the community for the field test. Participants were instructed on using the USSD code through an “information education and communication” (IEC) material developed by the NIMR I-TEST team. Each was provided with the OraQuick kit. Immediately after completing the test, participants were asked about their experience using the USSD platform.

### Data Analysis

The participants’ demographic characteristics (user testing and pilot study) were evaluated and reported as descriptive statistics. The recorded interview with the participants was transcribed verbatim, and inductive thematic analysis was done by members of the research team with experience in qualitative research. The inductive thematic analysis process ensured that the thematic analysis was completely data driven. The process involved repeated reading of the transcripts to become familiar with the data, generating initial codes relevant to the research questions, organizing the codes generated, arranging the subthemes into overarching themes, and defining and naming themes. A descriptive analysis of the findings from the heuristic questionnaire and the pilot study was also done. Variables were reported as proportions with bivariate analysis of the association between some variables through SPSS (version 26; IBM Corp).

### Ethics Approval

The institutional review board of the Nigerian Institute of Medical Research (IRB-18-028) and Saint Louis University (approval number 31457) gave ethical approval for the Innovative Tools to Expand HIVST study, and informed consent was obtained from each participant before the commencement of the usability beta testing study.

## Results

A total of 19 AYAs participated in the usability study. The median age was 19 (IQR 16-23) years. There were 11 females, 8 males, and 0 nonbinary individuals. They were all out-of-school AYAs.

### Usability Data

A total of 7 of Nielsen’s 10 usability heuristics yielded positive results. The visibility of the system status of the USSD platform showed that the user could tell the state of the platform and the alternatives for action (19/19), and the response times were appropriate to the user’s cognitive processing (18/19). The assessment of the match between the USSD platform and the real world showed that menu choices were ordered in the most logical way, given the user, the item names, and the task variables (18/19), and questions were stated in clear, simple language for the question-and-answer interfaces (19/19). Assessment of consistency and standards of the platform revealed that the field labels are consistent from 1 data entry screen to another (19/19), and commands are used the same way and mean the same thing in all parts of the system (16/19). Also, on whether the platform helps users to recognize, diagnose, and recover from errors, all the participants reported that messages place users in control of the system, and the prompts were brief and unambiguous. Similarly, the assessment of recognition and recall revealed that there was a good color and brightness contrast between image and background colors (19/19), and there was an obvious visual distinction made between the “choose one” menu and the “choose many” menus (18/19). On the aesthetic and minimalist design of the USSD platform, pop-up or pull-down menus within data entry fields do have many, but well-defined, entry options (19/19), and the field labels are brief, familiar, and descriptive (100%), while in the help and documentation assessment, all the users reported that instructions follow the sequence of user actions and the information on the platform is relevant (Table 1).

**Table 1:** Usability heuristics evaluation of the USSD platform by participants (n=19) among Nigerian youths, 2020.

Checklist		Yes, n (%)	No, n (%)
**Visibility of system status**			
	Does the system provide *visibility—*that is, by looking, can the user tell the state of the system and the alternatives for action?	19 (100)	0 (0)
	Are response times appropriate to the user’s cognitive processing?	18 (94.7)	1 (5.3)
**Match between system and the real world**			
	Are menu choices ordered in the most logical way, given the user, the item names, and the task variables?	18 (94.7)	1 (5.3)
	For question-and-answer interfaces, are questions stated in clear, simple language?	19 (100)	0 (0)
**User control and freedom**			
	Can users easily reverse their actions?	5 (26.3)	14 (73.7)
	If the system uses a question-and-answer interface, can users go back to previous questions or skip forward to later questions?	12 (63.2)	7 (36.8)
	If users can go back to a previous menu, can they change their earlier menu choice?	11 (57.9)	8 (42.1)
**Consistency and standards**			
	Are field labels consistent from 1 data entry screen to another?	19 (100)	0 (0)
	Are commands used the same way, and do they mean the same thing, in all parts of the system?	16 (84.2)	3 (15.8)
**Help users recognize, diagnose, and recover from errors**			
	Do messages place users in control of the system?	19 (100)	0 (0)
	Are prompts brief and unambiguous?	19 (100)	0 (0)
**Error prevention**			
	Does the system prevent users from making errors whenever possible?	13 (68.4)	6 (31.6)
	Does the system warn users if they are about to make a potentially serious error?	12 (63.2)	7 (36.8)
**Recognition rather than recall**			
	Is there good color and brightness contrast between the image and background colors?	19 (100)	0 (0)
	Is there an obvious visual distinction made between “choose one” and “choose many” menus?	18 (94.7)	1 (5.3)
**Aesthetic** **and minimalist design**			
	Are there pop-up or pull-down menus within data entry fields that have many, but well-defined, entry options?	16 (84.2)	3 (15.8)
	Are field labels brief, familiar, and descriptive?	19 (100)	0 (0)
**Help and documentation**			
	Do the instructions follow the sequence of user actions?	19 (100)	0 (0)
	Is the information relevant?	19 (100)	0 (0)
**Autonomy**			
	Does the system or device give the option to return to factory settings?	10 (52.6)	9 (47.4)
	Can the user take their own decisions? (Personalization)	16 (84.2)	3 (15.8)

However, 3 of Nielsen's usability heuristics yielded negative results. These were user control and freedom, error prevention, and autonomy. The assessment of the user’s control and freedom revealed that the majority of the participants felt that users could not easily reverse their actions (14/19), and 7 out of 19 felt that if the platform uses a question-and-answer interface, users cannot go back to previous questions or skip forward to later questions, and 8/19 felt that users cannot change their earlier menu choice if they can go back to a previous menu. Concerning error prevention, 6 out of 19 of the participants believe that the platform does not prevent users from making errors whenever possible, and 7 out of 19 felt that the platform does warn users if they are about to make a potentially serious error. While the assessment of user autonomy showed that 9 out of 19 participants believed that the platform does not give the option to return to factory settings.

Review and recommendations from the heuristic evaluation: 1 of the limitations of a USSD platform is the difficulty or challenge of reversing actions after they have been completed or when an error occurs while using the platform without an opportunity to go back to the beginning. It was recommended that the question prompts on the platform be simplified to reduce this challenge. Also, to address the concern about users' autonomy, it was recommended that a unique ID be assigned to the user instead of a name. These changes were incorporated into the pilot study.

### Exit Interview

A qualitative assessment of the user experience was done through exit interviews. A total of 13 youths out of the 19 who participated in the usability testing agreed to participate in the exit interview.

#### Ease of Use

The participants expressed various views on the ease of use of the USSD platform for verifying HIVST results and linkage to care. Most participants believed that the USSD platform was easy to use without any significant challenge in guiding users toward performing HIVST, interpreting the result, and getting access to a trained counselor about their test results. However, some participants had some challenges in using the platform because of connectivity issues arising from their mobile network operator.

It was easy to understand, has simple language, and it is efficient to say a few

it was cool, matches the trend of the social media age. This experience means a lot. However, a few participants did not find it that easy to use mainly because of mobile network issues which were due to their GSM service provider.

#### Personal Preference About the Unstructured Supplementary Service Data Platform

When asked about their personal preference regarding the platform, about half of the respondents did not have any particular preference for the USSD. However, a few participants submitted that they were impressed by the simplicity of the question prompts on the USSD platform. Another participant stated that she prefers the fact that it is easily accessible and a good alternative to going to the hospital for HIV tests, as it promotes privacy when performing HIVST. One participant, however, had a concern about the fact that the USSD platform asks for some personal or sensitive information.

It was asking for basic information and I wondered the need for that

It is easily accessible, rather than going to the hospital. Especially the youths who spend most of their time with their phones.

#### The Aspect of the Unstructured Supplementary Service Data Platform That Needs Improvement

Some of the participants noted that the USSD platform would not work when the required code is dialed if there is no phone call credit on some of the mobile network operators’ platforms in Nigeria. However, it appears that this challenge is outside the immediate control of the study investigator. Otherwise, the majority of the participants felt there was no serious need for a significant improvement to the USSD platform in its current state.

#### Referral After Test

All the participants reported that they are happy with the feature of the USSD platform that provides linkage to experienced AYA counselors through phone calls after performing and interpreting their HIVST results, and all unanimously submitted that they would recommend this USSD code or platform to their friends who would like to do HIV self-testing and who do not have a smartphone.

### Pilot Field Test

Pilot field testing of the USSD platform was done among 164 young persons. Females were in the majority (101/164, 61.6%), and the mean age of the participants was 17.5 (SD 3.18) years. Most participants were out-of-school AYAs involved in various unskilled and semiskilled work, trading, and nonprofessional work in the communities. Evaluation of their sexual orientation revealed that 92.1% (151/164) of the participants reported that they were heterosexual, while 7.9% (13/164) of them reported that they were gay (Table 2). All the participants in the pilot study were able to conduct HIVST, interpret their results, and use the linkage to care feature of the USSD platform without any challenge. A total of 7.9% (13/164) of youths had reactive HIV results (reactive to the OraQuick kit), and 92.1% (151/164) of them were nonreactive. However, among the 13 persons who were reactive and came for a confirmatory test at NIMR, none were confirmed HIV positive.

**Table 2:** Sociodemographic characteristics of the field trial participants (N=164) among Nigerian youths, 2020.

Variable		Frequency (N=164), n (%)
**Sex**		
	Male	101 (61.6)
	Female	63 (38.4)
**Sexual orientation**		
	Heterosexual	151 (92.1)
	Same-sex attracted	13 (7.9)
**HIV test result**		
	Nonreactive	151 (92.1)
	Reactive	13 (7.9)
Age (years), mean (SD)		17.5 (3.18)

There was no statistically significant association between the gender of the participants and the HIV-positive result done with the test kit (*χ*^2^_1_=0.4; *P*=.76). Also, older participants had positive results using the test kit with a mean age of 19 (SD 3.2) years compared to those who were negative with a mean age of 17 (SD 3.2) years. This difference in age and the HIV-positive test result was not statistically significant (*t*_1_=1.60; *P*=.11). However, a statistically significant association existed between participants’ sexual orientation and HIV-positive test results using the OraQuick test kits. A total of 38% of persons who identified as gay were reactive using the test kit when compared to 5.3% among those who were heterosexual (*χ*^2^_1_=18; *P*<0.001; Table 3).

**Table 3:** Association between participants’ sociodemographic variables and positive HIV results (N=164) among Nigerian youths, 2020.

Variables			Nonreactive, n (%)		Reactive, n (%)		Chi-square (*df*)	*t* test (*df*)		*P* value
**Sex**							0.4 (1)	N/A^a^		.76
	Male	92 (91.1)		9 (8.9)						
	Female	59 (93.7)		4 (6.3)						
**Sexual orientation**							18 (1)	N/A		.001
	Heterosexual	143 (94.7)		8 (5.3)						
	Same-sex attracted	8 (61.5)		5 (38.5)						
Age (years), mean (SD)			17.38 (3.16)		18.85 (3.21)		N/A	1.60 (1)		0.11

^a^N/A: Not applicable.

## Discussion

### Overview

This study reported on the development, usability, and field pilot of a USSD system to verify HIV self-testing results among AYAs in Nigeria. The study demonstrated the benefits of involving AYAs in the development and usability testing of an intervention aimed at addressing a health challenge peculiar to them. It extends the existing literature [2,14,24] on the feasibility of the use of mHealth among AYAs in Africa. From the study, we were able to demonstrate that the usability of the USSD platform as an alternative platform to the use of mobile apps in the verification of HIV self-testing among young people is good and well accepted.

The young persons who participated in the exit interviews reported that the platform was easy to use and had no significant challenges except for the occasional inability to access the USSD service on their phones, mainly due to mobile network service interruptions. This was said to occur when the user does not have call credit on their mobile phone. This is similar to a challenge with mobile network operators reported by a South African study that submitted that the USSD is prone to network and user timeouts [25].

One of the important features of the USSD platform in interpreting the result of HIVST and promoting linkage to care is that users of the platform could indicate how they would prefer to be contacted after performing HIVST. This could be either to understand the test result or to learn more about HIV prevention, in which case they could either call a mobile phone number or request a call from a trained AYA counselor.

The participants in the heuristic evaluation of the USSD platform reported 3 issues regarding the ease of reversing an action or making corrections if an error was committed when using the platform and the perceived challenge of the user’s autonomy. Though modifications were made to reduce these challenges in the final product, it is noteworthy that these were some of the challenges associated with using the USSD platform for health interventions, as reported by previous studies [25]. However, the benefits of improved health and access provided by the USSD platform were thought to outweigh these challenges [26].

We believe usability assessment using heuristic evaluations among potential end users is unique to this study because the traditional approach for heuristic evaluation uses few content experts, with its attendant shortcomings. However, as previously documented by Silverbratt et al [27], heuristic evaluations involving end users have a higher frequency and relevance value, and the potential of real-life assessment of a new app toward improved acceptability of the same [27].

The proportion of participants in the pilot study who tested positive after HIVST was about 7.9% (13/164), and none were confirmed to be living with HIV infection. This finding was not surprising because a previous study documented the sensitivity of the Oraquick HIVST kit to be between 92% and 93%, even though its specificity is as high as 99.9% [28,29]. Expectedly, most of the participants in the pilot study requested a phone call from the study team through the USSD platform after their HIVST was completed. All participants with reactive HIV test results were contacted by the AYA counselor and encouraged to do confirmatory HIV testing.

Furthermore, given that all categories of young people could benefit from the use of the USSD platform for HIVST because it is cheaper, and does not require the use of internet data, and can be used on all mobile phones (whether they are smartphones or not), we chose the population of out-of-school young persons who were not likely to have a smartphone in the inner-city of an urban area of Lagos State. Successful piloting of the USSD platform among this population suggests that the USSD platform would be useful in promoting HIVST among many Nigerian AYAs. Also, the iterative design that used input from end users in the design increased the acceptability of the platform in promoting HIVST.

Even though the piloting was done in an urban population among out-of-school young persons, caution needs to be expressed in generalizing the findings to the population of AYAs in Nigeria. Also, though the exit interviews were conducted with measures to ensure privacy, removing the potential for social desirability bias is difficult because the young people might want to say what they consider appropriate about using a new mobile phone feature.

### Conclusions

This study demonstrated the usability and feasibility of the USSD system as an alternative to mobile phone apps for the verification of HIVST results among the population of Nigerian AYAs without access to smartphones. Hence, the scale-up of access to creative and youth-friendly mHealth interventions is urgently needed, especially among the AYA population, who would benefit from the USSD for HIVST services. Additionally, further research using implementation research methods is necessary to further document this evidence-based intervention’s adoption, feasibility, and sustainability in resource-limited settings.

## Appendix

Multimedia Appendix 1 Unstructured supplementary service data (USSD) instruction flow for HIV self-testing (HIVST).

Multimedia Appendix 2 Administrative dashboard of the unstructured supplementary service data (USSD) app.

## Abbreviations

AYA: adolescents and young adult
CDA: community development association
HIVST: HIV self-testing
IEC: information education and communication
I-TEST: innovative tools to expand HIVST
LMIC: low- and middle-income country
mHealth: mobile health
NIH: National Institutes of Health
NIMR: Nigerian Institute of Medical Research
SRH: sexual and reproductive health
STI: sexually transmitted infection
USSD: unstructured supplementary service data

## Data Availability

The data sets generated during and analyzed during this study are available from the corresponding author on reasonable request.
