# Supplementary figures and images for: An Unstructured Supplementary Service Data System to Verify HIV Self-Testing Among Nigerian Youths: Mixed Methods Analysis of Usability and Feasibility
Source: JMIR Form Res. 2023 Sep 25;7:e44402. doi: 10.2196/44402 (PMC10562967; doi:10.2196/44402)

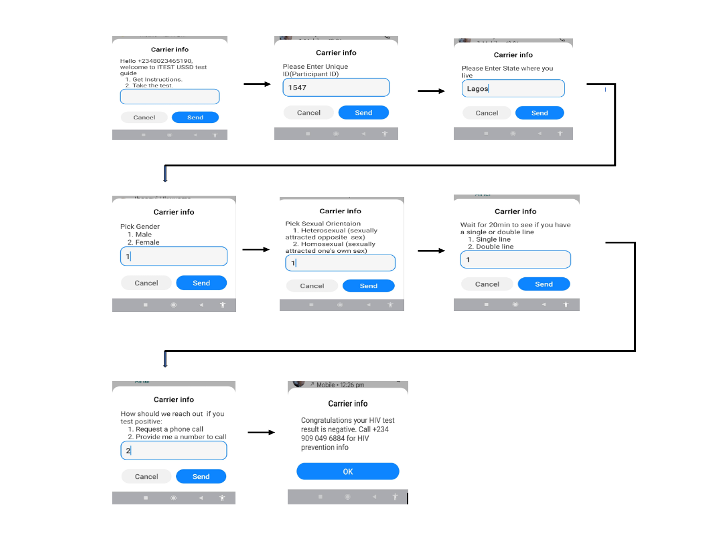

Supplement: Multimedia Appendix 1 [file formative_v7i1e44402_app1.png]

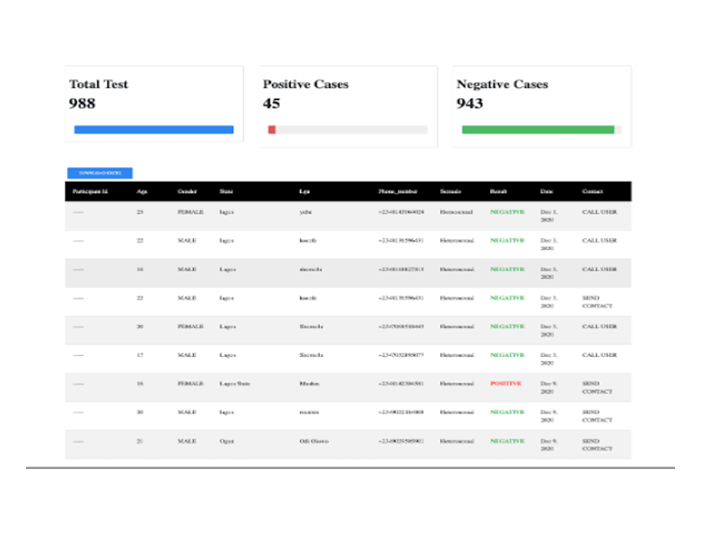

Supplement: Multimedia Appendix 2 [file formative_v7i1e44402_app2.png]
